# The NIa-Protease Protein Encoded by the *Pepper Mottle Virus* Is a Pathogenicity Determinant and Releases DNA Methylation of *Nicotiana benthamiana*

**DOI:** 10.3389/fmicb.2020.00102

**Published:** 2020-02-21

**Authors:** Yi-Nuo Gong, Ru-Qing Tang, Yu Zhang, Jing Peng, OuYang Xian, Zhan-Hong Zhang, Song-Bai Zhang, De-Yong Zhang, Hui Liu, Xiang-Wen Luo, Yong Liu

**Affiliations:** ^1^Longping Branch, Graduate School of Hunan University, Changsha, China; ^2^Hunan Academy of Agricultural Sciences, Changsha, China; ^3^National Agro-Tech Extension and Service Center, Beijing, China

**Keywords:** NIa-Pro, DNA methylation, transcriptional gene silencing, *Pepper mottle virus*, pathogenicity determinant

## Abstract

It is well documented that the canonical function of NIa-protease (NIa-Pro) of the potyviruses is responsible for cleaving the viral polyprotein into functional proteins. Although NIa-Pro is vital for the infection cycle of potyviruses, the function of NIa-Pro in the interaction of the potyvirus host is not clear. In this study, NIa-Pro is ectopically expressed from a *potato virus X* (PVX) vector and infiltrates *Nicotiana benthamiana* wild type and 16-TGS. The pathogenicity and inhibition of host transcriptional gene silencing (TGS) are characterized. Ectopic expression of NIa-Pro from a PVX vector resulted in severe mosaic symptoms followed by a hypersensitive-like response in *N. benthamiana*. Furthermore, PepMoV NIa-Pro was able to reverse established TGS of a green fluorescent protein transgene by reducing methylation of promoter sequences in *N. benthamiana* and possessed the capacity to interfere with the global methylation of *N. benthamiana*. Taken together, the results of this study likely suggest that PepMoV NIa-Pro is a pathogenicity determinant and a potent suppressor of host TGS and suggest that NIa-Pro may employ novel mechanisms to suppress host antiviral defenses. To the best of our knowledge, this is the first report of a plant RNA virus modulating host TGS in a novel manner by interfering with the establishment of the methylation step of the plant DNA methylation pathway.

## Introduction

Potyviruses (genus *Potyvirus*, family *Potyviridae*) constitute the largest group of plant-infecting RNA viruses affecting major crops worldwide. Potyviruses encode a large polyprotein that is subsequently cleaved by three virus-encoded proteinases to yield up to 10 mature proteins (Shukla et al., 1994). Additionally, a P3N–PIPO is a fusion of the N-terminal part of P3 and the PIPO protein-coding regions by polymerase slippage (Rodamilans et al., 2015; Untiveros et al., 2016). The main viral proteinase, NIa, is a polyprotein consisting of two domains, viral protein genome-linked (VPg) and protease (NIa-Pro), separated by an inefficiently utilized self-proteolytic site. NIa-Pro encoded by potyviruses plays many roles during the virus infecting cycle. The canonical function of NIa-Pro is the main protease of the virus responsible for the cleavage of most sites in the polyprotein (Sun et al., 2010). Tobacco etch potyvirus encoding NIa-Pro can bind NIb, which is critical for *tobacco etch virus* (TEV) genome replication (Daros et al., 1999). Further proteomic studies demonstrated that TEV NIa-Pro could interact with 76 host proteins involved in response to host stress, metabolism, and photosynthesis (Martínez et al., 2016). Additionally, NIa-Pro induced vein necrosis in tobacco by *potato virus Y* (PVY) isolates (Faurez et al., 2012), which indicated that NIa-Pro of the potyviruses may be a pathogenicity determinant.

Cytosine DNA methylation, one of the fundamental aspects of stable epigenetic modification, modulates coding genes that involve various cellular biological processes and molecular functions to counter virus infection (Wang et al., 2019). The cytosines in the genome of DNA viruses could be directly methylated through plant DNA methylation; for example, geminivirus transcription is usually disrupted by host DNA methylation, which occurs in the viral genome through RNA-directed DNA methylation (RdDM) (Coursey et al., 2018). Although RNA viruses cannot be directly methylated by plant DNA methylation owing to the absence of DNA in the replication process, the 2b protein encoded by *cucumber mosaic virus* (CMV) could bind the AGO4-related small RNAs and impairs AGO4 activities to release the DNA methylation of *Nicotiana benthamiana*, suggesting the modulation of RNA virus replication by plant DNA methylation (Hamera et al., 2012).

To counter the suppression of viral transcription mediated by plant DNA methylation, DNA viruses, mainly from geminiviruses, encode DNA methylation suppressor proteins. The C2 and C4 proteins encoded by *tomato leaf curl Yunnan virus* (TLCYnV), the V2 protein encoded by *Tomato yellow leaf curl virus* (TYLCV), and the βC1 and the Rep proteins encoded by geminiviruses can inhibit host transcriptional gene silencing (TGS) and modulate the plant DNA methylation pathway (Yang et al., 2011; Zhang et al., 2011; Rodrıguez-Negrete et al., 2013; Xie et al., 2013; Wang et al., 2014). However, the DNA methylation suppressor proteins encoded by plant RNA viruses have little been documented until now (Hamera et al., 2012).

PepMoV was first identified as an atypical species of potyvirus (Nelson and Wheeler, 1972) and is known to cause serious symptoms in many solanaceous plants (Han et al., 2006). Although most proteins encoded by potyviruses have been well characterized, the functions of PepMoV-encoded proteins are mostly annotated through comparison with other species of potyviruses and remain to be identified.

In this study, the functions of PepMoV-encoded NIa-Pro were characterized by ectopic expression of *NIa-Pro* from a *potato virus X* (PVX) vector, and the results demonstrated that PepMoV NIa-Pro is a pathogenicity determinant and a potent suppressor of host TGS and suggest that NIa-Pro may employ novel mechanisms to suppress host antiviral defenses.

## Materials and Methods

### Plant Materials

Wild-type and 16-TGS lines (Buchmann et al., 2009) of *Nicotiana benthamiana* Domin were used in this study. Plants were grown inside a culture room set at 25 ± 2°C and 60% relative humidity with a 16-h light/8-h dark illumination. The third or fourth leaves of 6- to 8-week-old plants were infiltrated individually with one of the agrobacterium cultures containing the plasmid made in this study.

### Plasmid Constructs and Agroinfiltration

The *NIa-Pro* gene sequence was cloned from PepMoV isolate HN (GenBank accession No. LN832375) (Zhang et al., 2019) and inserted into a PVX-based vector (pGR106) between the *Cla*I and *Sal*I sites. The recombinant pGR106 vectors expressing the *NIa-Pro* gene or β*C1* gene [as a positive control, cloned from *tomato yellow leaf curl China virus betasatellite* (TYLCCNB) and described previously] (Buchmann et al., 2009) were transformed into *Agrobacterium tumefaciens* GV3101. The *Agrobacterium* cultures were cultured in Luria broth with appropriate antibiotic selection at 28°C for 16 h. The cells were centrifugated and resuspended in infiltration medium (10 mM of MgCl_2_ and 20 mM of acetosyringone) to an OD600 0.4 to 0.6 and incubated at room temperature for 3 h before injection into leaves. The underside of the third or fourth leaf of *N. benthamiana* was infiltrated with syringe (Rajamaki and Valkonen, 2009).

### H_2_O_2_ Detection

The production and accumulation of H_2_O_2_ was detected visually in leaves using the 3,3′-diaminobenzidine (DAB)–HCl (Sigma) uptake method as described previously (Sharma et al., 2010).

### DNA Methylation Analysis Using Restriction Digestion and Quantitative Real-Time PCR

Genomic DNA was extracted from plant leaves using the cetyl trimethylammonium bromide (CTAB) reagent (Tiangen, Beijing, China). *Dra*I (TaKaRa, Kyoto, Japan), a methylation-insensitive restriction endonuclease, and *Mcr*BC (New England Biolabs, Ipswich, MA, United States). A methylation-dependent restriction endonuclease was used for digestion analysis of genomic DNA according to the manufacturer’s instructions. Briefly, genomic DNA (10 ng) was digested by 50 U of the respective enzyme in a 50-ml reaction mix. Digested genomic DNA was immediately separated by 1.5% agarose gel electrophoresis. For quantitative real-time PCR (qPCR) analysis, specific primer pairs for 35S promoter or *GFP* gene (Supplementary Table S1) were used, and *NbActin* was set as reference gene (Li et al., 2015).

### RNA Extraction and Reverse Transcription Quantitative Real-Time PCR Analysis

Total RNA was isolated from infiltrated leaves of wild-type or 16-TGS *N. benthamiana* using TRIzol reagent (Tiangen, Beijing, China). For reverse transcription quantitative real-time PCR (RT-qPCR) analysis, specific primer pairs (Supplementary Table S1) were designed by Primer Premier 5 software or referred to published documents, and *NbGADPH* was set as reference gene (Li et al., 2014).

### Western Blotting

Total protein was extracted from infiltrated systemic leaves as described previously (Li et al., 2014). Western blotting was performed with primary mouse monoclonal antibody [green fluorescent protein (GFP) or PVX CP protein monoclonal antibody, HuaAn Beijing, China], followed by goat anti-mouse secondary antibody conjugated to alkaline peroxidase (Bio-Rad). Blotted membranes were washed thoroughly and visualized using NBT/BCIP.

### Green Fluorescent Protein Images

To visualize GFP in TGS assays, representative plants were photographed at 14 dpi with a Canon 400D digital camera with a 58-mm yellow filter. GFP fluorescence was activated by a hand-held longwave UV lamp (100 W, Black Ray model B 100 A; UV products).

## Results

### Phylogenetic Analyses of PepMoV-Encoded NIa-Pro

**FIGURE 1:**
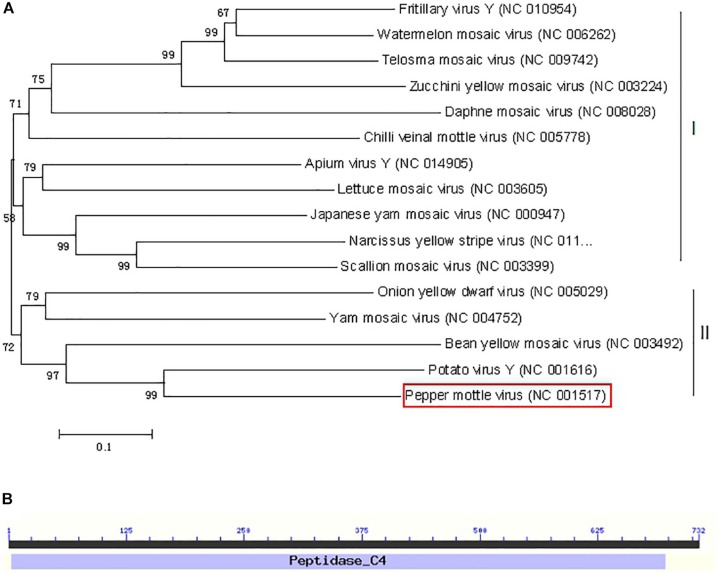
Phylogenetic relationships and domain structure of NIa-Pro nucleotide (nt) sequences of representative potyviruses (*NIa-Pro* gene of *Pepper mottle virus* shown in red box. **(A)** The nucleotide sequences of the NIa-pro gene from 16 potyvirus viruses were aligned using the neighbor-joining method with the MEGA6 program with 1,000 replications. The numbers beside each node represent the percentages for the bootstrap value (>50%). **(B)** One conserved peptidase domain was identified using the Conserved Domain Database (CDD).

To examine the evolutionary relationships of NIa-Pro sequences from different potyviruses, a phylogenetic dendrogram of the complete NIa-Pro nucleotide sequences of 16 representative potyvirus species was generated. As shown in Figure 1A, two major subclusters are grouped. Subcluster I contains 11 of 16 potyviruses, and cluster II consists of five of 16 potyviruses. The PepMoV NIa-Pro protein is located in cluster II and shares the highest sequence identity with PVY. Sequence alignment showed that potyvirus NIa-Pro proteins are highly divergent in amino acid sequences. The structure of the NIa-Pro protein was analyzed by the Conserved Domain Database (CCD) available at the National Center for Biotechnology Information (Marchler-Bauer et al., 2011), and only one conserved domain architecture was retrieved for the NIa-Pro protein (Figure 1B). All NIa-Pro proteins from potyviruses contain this conserved domain. The presence of the same conserved position in many potyviruses suggests that NIa-Pro may play an important role during virus infection.

### NIa-Pro Is a Pathogenicity Determinant in *Nicotiana benthamiana*

**FIGURE 2:**
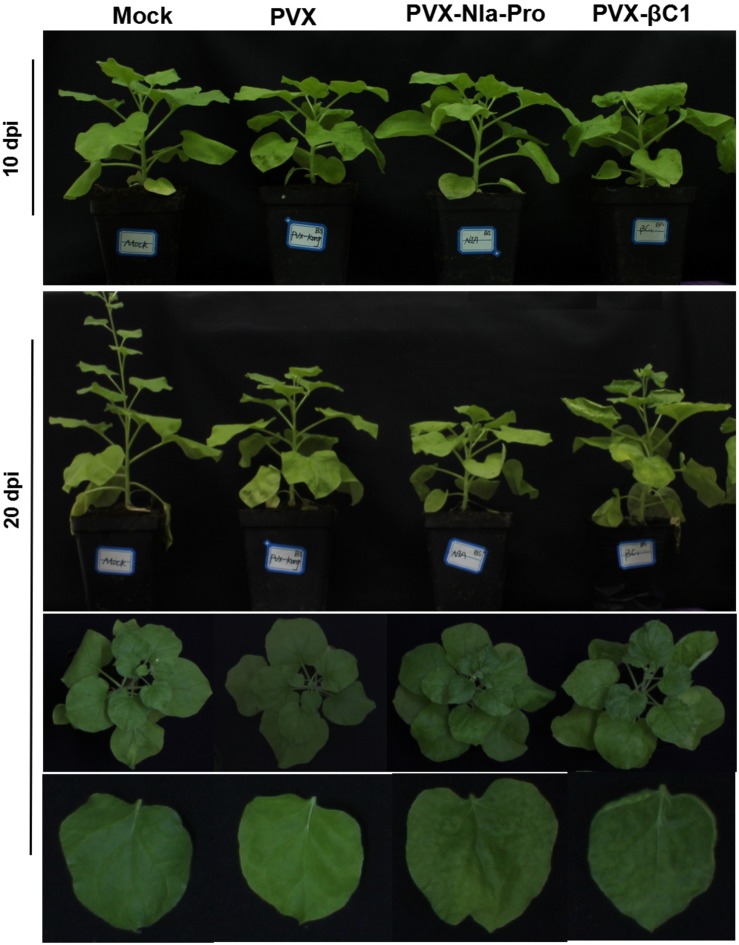
Symptoms exhibited by plants following inoculation with PVX-NIa-Pro.

To determine whether ectopically expressed NIa-Pro is a symptom determinant in *Nicotiana benthamiana*, the *NIa-Pro* gene was inserted into a PVX-based vector (pGR106) for overexpression. As shown in Figure 2 (upper panel), the phenotype in plants infected with PVX was similar to that of plants infected with PVX-NIa-Pro at 10 dpi. At 20 dpi, PVX-infected plants showed a loss of veinal chlorosis and mild mosaic symptoms; however, continuous NIa-Pro expression resulted in symptoms of severe mosaic, chlorosis, and leaf deformity at 20 dpi (Figure 2, lower panel). In addition, pronounced necrotic symptoms were observed on the systemic leaves of PVX-βC1-infected plants, but plants inoculated with PVX-NIa-Pro failed to develop the necrotic phenotype (Figure 2, lower panel).

### NIa-Pro Elicits a Hypersensitive-Like Response in *Nicotiana benthamiana*

**FIGURE 3:**
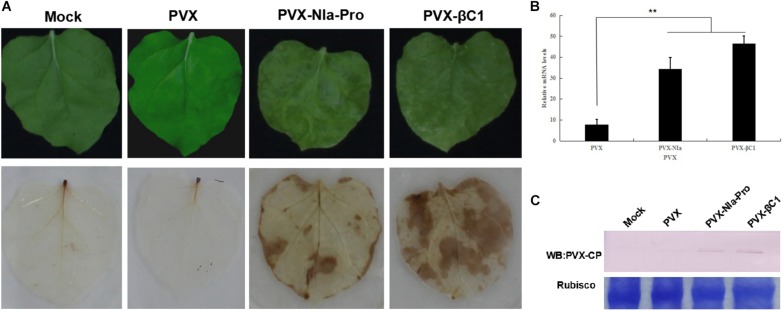
Symptoms exhibited H_2_O_2_ accumulation in plants inoculated with *potato virus X* (PVX) or PVX-NIa-Pro. **(A)** Severe mosaic symptoms and H_2_O_2_ accumulation induced by PVX-NIa-Pro. *Nicotiana benthamiana* plants were infected with PVX or PVX-NIa-Pro. Upper infected leaves with 3,3’-diaminobenzidine (DAB) staining (lower panel) were photographed directly at 20 dpi. **(B)** RT-qPCR analysis of PVX genomic RNA in systemic leaves from *N. benthamiana* plants infected with PVX or PVX-NIa-Pro at 20 dpi. Values are the mean ± SD. Highly significant differences (** *p* < 0.01) between samples in each pair are indicated. **(C)** Western blotting analysis of PVX CP protein in systemic leaves from *N. benthamiana* plants infected with PVX or PVX-NIa-Pro at 20 dpi.

To determine whether NIa-Pro elicits the hypersensitive-like response (HLR), PVX-, and PVX-NIa-Pro-infected leaves were analyzed by the DAB uptake method. DAB polymers can stain the accumulated H_2_O_2_ as a deep brown color, which can be visualized after ethanol clearing of the tissue. As anticipated, compared with PVX-infected plants, PVX-NIa-Pro-infected plants accumulated high concentrations of H_2_O_2_ in systemically infected leaves (Figure 3A). To determine whether severe symptoms in the presence of NIa-Pro are a consequence of higher accumulation of PVX, RT-qPCR and Western blotting against PVX CP protein were employed to examine PVX genomic RNA levels and accumulation of CP protein at 20 dpi (Figures 3B,C). More PVX genomic RNAs and CP proteins accumulated in PVX-NIa-Pro-infected plants compared with PVX-infected plants at 20 dpi, suggesting that NIa-Pro is a virulence factor that enhances replication of PVX in *N. benthamiana*. These data reveal that NIa-Pro is a symptom determinant that can elicit an HLR when expressed from PVX-NIa-Pro.

**FIGURE 4:**
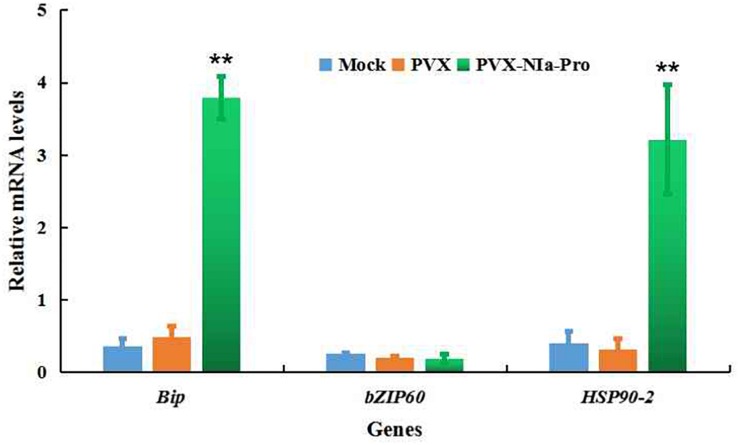
Relative mRNA levels of the indicated endoplasmic reticulum (ER) stress marker genes in NIa-Pro-expressing *Nicotiana benthamiana* plants as determined by qRT-PCR. Relative mRNA levels of *N. benthamiana ER-localized binding* (*Bip*), *Heat shock 90-2* (*HSP90-2*), and *basic leucine zipper 60* (*bZIP60*) were measured in *potato virus X* (PVX)-NIa-Pro and PVX vector-infected *N. benthamiana* plants at 20 days post inoculation (dpi). Values are the mean ± SD. Highly significant differences (** *p* < 0.01) between samples in each pair are indicated.

To test if there was any association between NIa-Pro and endoplasmic reticulum (ER) stress, the expression levels of key genes involved in ER stress, including *Bip* (ER-localized binding protein), *HSP90-2* (heat shock protein 90-2), and *bZIP60* (basic leucine zipper protein 60), were quantified. qRT-PCR analysis showed that the expression of *Bip* and *HSP90-2* was significantly induced by NIa-Pro; otherwise, the expression of *bZIP60* was not influenced by NIa-Pro (Figure 4). These results suggest that NIa-Pro can induce ER stress by stabilizing proteins for correct folding or re-folding in response to ER stress but not the unfold protein response (UPR) to ER stress.

### NIa-Pro Reverses Established Methylation-Mediated Transcriptional Gene Silencing

**FIGURE 5:**
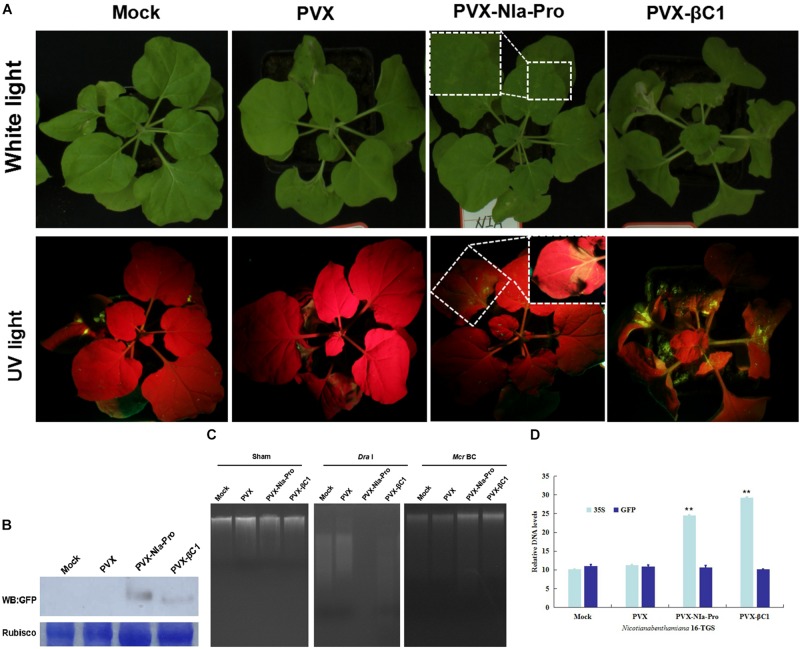
NIa-Pro inhibits transcriptional gene silencing (TGS) of a green fluorescent protein (GFP) transgene and suppresses DNA methylation. **(A)** *Nicotiana benthamiana* 16-TGS plants were mock-inoculated or inoculated with *potato virus X* (PVX), PVX-NIa-Pro, or PVX-βC1 (positive control), and leaves were photographed under white light or UV light at 14 days post inoculation (dpi). **(B)** Western blotting assay of GFP protein accumulation in systemically infected leaves shown in panel **(A)**. Coomassie blue staining of the large subunit of Rubisco was used as loading controls for Western blotting. **(C)** DNA methylation analysis by restriction enzyme digestion in NIa-Pro-expressing *N. benthamiana* plants. Genomic DNA extracted from systemically infected leaves shown in **(A)** was digested with the methylation-dependent restriction enzyme *Mcr*BC and the methylation-insensitive enzyme *Dra*I. “Sham” indicates a mock digestion with no enzyme included. **(D)** qPCR analysis of DNA levels from systemically infected leaves shown in panel **(A)** was digested with the methylation-dependent restriction enzyme *Mcr*BC. Values are the mean ± SD. Highly significant differences (** *p* < 0.01) between samples in each pair are indicated.

Plant DNA methylation modulates coding-gene expression to induce TGS (Law and Jacobsen, 2010). To determine whether NIa-Pro inhibited plant methylation-mediated TGS, transgenic *N. benthamiana* 16-TGS plants containing a GFP transgene downstream of a transcriptionally silenced CaMV 35S promoter were employed in this study. Previously, this assay demonstrated the TGS suppression activity of several geminivirus-encoded proteins (Raja et al., 2008; Yang et al., 2011). The mock culture (GV3101 *Agrobacterium tumefaciens* cultures), PVX (negative control), PVX-βC1 (positive control), or PVX-NIa-Pro 16-TGS plants were inoculated in the third and fourth leaves. By 14 dpi, mild symptoms were exhibited in systemic leaves of PVX-infected plants; however, the typical βC1-associated phenotypes, including enations and upward leaf curling, developed in systemic leaves of PVX-βC1-infected plants. The phenotypes induced by PVX and PCX-βC1 were in agreement with those described previously (Yang et al., 2011). Severe mosaic symptoms developed in plants infected with PVX-NIa-Pro. In addition, yellow-green GFP fluorescence was visible in plants infected with PVX-βC1 and PVX-NIa-Pro (Figure 5A). As anticipated, Western blot analysis verified the visible fluorescence in PVX-NIa-Pro- or PVX-βC1-inoculated plants (Figure 5B).

To determine whether NIa-Pro affects global DNA methylation, a methylation-dependent restriction enzyme (*Mcr*BC), which only cleaves methylated DNA sequences (Stewart et al., 2000), was used. Genomic DNA was extracted from leaves of *N. benthamiana* plants inoculated with mock culture (GV3101 *A. tumefaciens* cultures), PVX (negative control), PVX-βC1 (positive control), or PVX-NIa-Pro and subjected to restriction digestion. A mock digestion with no enzyme was included (sham) as a control. All four genomic DNA samples were able to digest by *Dra*I, a methylation-independent restriction endonuclease, as indicated by the presence of a “smear” of different sized fragments on the gel (Figure 5C). However, although *Mcr*BC was able to cleave a significant portion of the genomic DNA of plants with Mock and PVX into small fragments, DNA from plants expressing NIa-Pro and βC1 was largely resistant to *Mcr*BC digestion (Figure 5C). DNA quantification by qPCR of 35S promoter and adjacent *GFP* gene has shown that the DNA level of 35S of PVX-NIa-Pro was relative higher than that of mock and PVX; otherwise, no difference appeared in adjacent *GFP* gene (Figure 5D). Thus, the expression of NIa-Pro in *N. benthamiana* plants likely reduced DNA methylation at a genome-wide level.

### NIa-pro Expression Inhibits Transcription of *NbAGO4*

**FIGURE 6:**
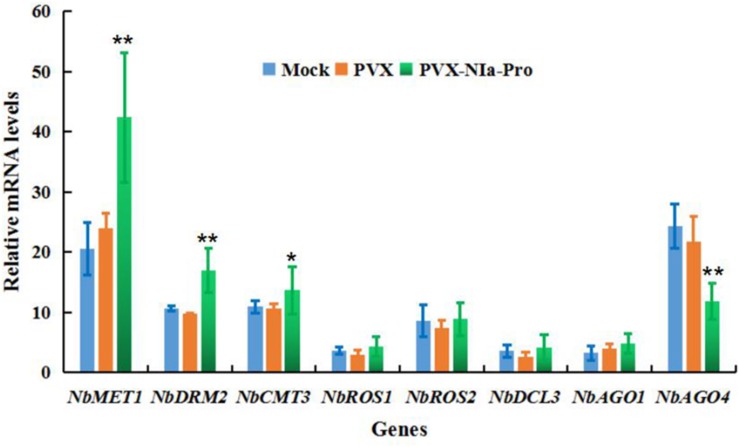
Overexpression of NIa-Pro represses transcription of *Nicotiana benthamiana* ARGONAUTE 4 (NbAGO4) mRNA. The effects of NIa-Pro on the expression of DNA methyltransferases, demethylases, and key genes involved in RNA-directed DNA methylation (RdDM) were analyzed by RT-qPCR. Relative mRNA levels of *N. benthamiana DNA METHYLTRANSFERASE1* (*NbMET1*), *NbDRM2*, *CHROMOMETHYLASE3* (*NbCMT3*), *REPRESSOR OF SILENCING 1* (*NbROS1*), *NbROS2*, *DICER3* (*NbDCL3*), *ARGONAUTE 1* (*NbAGO1*), and *NbAGO4* were measured in *potato virus X* (PVX)-NIa-Pro and PVX vector-infected *N. benthamiana* plants at 15 days post inoculation (dpi). Each mean value was derived from nine plants in three independent experiments. Student’s *t*-test was performed to assess differences between PVX-NIa- and PVX vector-infected plants. Values are the mean ± SD. Significant differences (* *p* < 0.05) and highly significant differences (** *p* < 0.01) between samples in each pair are indicated.

To provide mechanistic insights into the effects of NIa-Pro on the suppression of DNA methylation and epigenetic TGS, the expression levels of *N. benthamiana* DNA methyltransferases, demethylases, and key genes involved in RdDM in the presence of NIa-Pro were quantified by RT-qPCR. These genes included *N. benthamiana* homologs of *METHYLTRANSFERASE1* (*NbMET1*), *DRM2* (*NbDRM2*), *CHROMOMETHYLASE3* (*NbCMT3*), *REPRESSOR OF SILENCING1* (*NbROS1*), *REPRESSOR OF SILENCING 2* (*NbROS2*), *DICER3* (*NbDCL3*), *ARGONAUTE 1-1* (*NbAGO1*), and *ARGONAUTE 4-1* (*NbAGO4*). *N. benthamiana* plants expressing empty vector (PVX) and NIa-Pro were analyzed at 15 dpi. As shown in Figure 6, overexpression of NIa-Pro significantly induced *NbAGO4* expression (32% of that of control plants; *p* < 0.01), and expression of *NbMET1*, *NbDRM2*, and *NbCMT3* increased 46% (*p* < 0.01), 38% (*p* < 0.01), and 26% (*p* < 0.05), respectively (Figure 6). *NbROS1* and *NbROS2* expression increased by 18 and 15%, respectively, but not significantly (Figure 6). Taken together, these results demonstrate that the expression of NIa-Pro significantly suppresses the expression of *NbAGO4*, a gene responsible for establishing DNA methylation, which is the first step of the plant DNA methylation pathway. In addition, activation of *NbROS1* and *NbROS2*, two DNA glycosylases and repressors of TGS, may also contribute to TGS suppression by NIa-Pro.

## Discussion

PepMoV was first identified as an atypical isolate of the genus *Potyvirus* for serum differentiation and low amino acid sequence identity (Zitter, 1972). For example, PepMoV NIa-Pro exhibits 63.5% identity in sequence with PVY NIa-Pro and only 45.9% identity with TEV NIa-Pro (Mestre et al., 2000). Although the canonical function of NIa-protease (NIa-Pro) encoded by all potyviruses is responsible for cleaving the viral polyprotein into functional proteins, the other functions of NIa-Pro in the virus infecting cycle of potyviruses are inconsistent. TEV NIa-Pro could not induce a hypersensitive response (HR); however, PVY NIa-Pro could induce an HR in *Ry-*mediated resistance potatoes (Mestre et al., 2000). PepMoV NIa-Pro shared the highest nucleotide and amino acid sequences with PVY (Figure 1), and PVY NIa-Pro induced tobacco vein necrosis with HR (Faurez et al., 2012), suggesting PepMoV NIa-Pro would be a pathogenicity determinant. In this study, by using a PVX vector as an ectopic expression system, we found that PepMoV NIa-Pro could result in severe mosaic symptoms (Figure 2) and enhanced PVX replication (Figures 3B,C) but was not able to induce vein necrosis in leaves in *Nicotiana benthamiana*, which demonstrated that PepMoV NIa-Pro is a symptom determinant; however, it functioned in a different manner with PVY NIa-Pro.

Plant responses to infection by pathogens are often accompanied by a HLR, which is associated with an oxidative burst (including O_2_ and H_2_O_2_) (Bao and Howell, 2017). PepMoV NIa-Pro can induce H_2_O_2_ accumulation during infection with a PVX-based vector expressing NIa-Pro (Figure 3A). Additionally, PepMoV NIa-Pro could significantly activate genes expressing *Bip* and *HSP90-2* but did not change the expression of *bZIP60* (Figure 4). *Bip* is a chaperone gene that restores proper protein folding during ER stress (Bao and Howell, 2017), the *HSP90-2* gene is involved in stabilizing proteins for correct folding or re-folding in response to ER stress (Sangster et al., 2007), and *bZIP60* is the downstream transcription factor that activates genes required for coping with UPR (Arias Gaguancela et al., 2016). Therefore, PepMoV NIa-Pro likely induces *N. benthamiana* ER stress with a branch pathway of protein folding but does not activate the UPR.

It was well documented that DNA viruses, such as geminivirus-encoded pathogenicity determinants, also function as TGS suppressors targeting different steps of the TGS pathway. The βC1 protein of TYLCCNB directly interacts and inhibits host *S*-adenosyl homocysteine hydrolase (SAHH) activity to suppress host DNA methylation (Yang et al., 2011). The C2 protein of *beet curly top virus* (BCTV) attenuates the degradation of *S*-adenosyl-methionine decarboxylase 1 (SAMDC1) of *Arabidopsis* to inhibit host DNA methylation (Zhang et al., 2011). Intriguingly, a recent study demonstrated that the pathogenicity determinant of RNA viruses could also modulate the host methylation pathway. The 1a protein encoded by CMV, which is essential for viral infection, could recruit a novel methyltransferase from *Nicotiana tabacum* to methylate itself, which promotes CMV infection (Kim et al., 2008). Our results showed that PepMoV NIa-Pro can reverse the established TGS of *N. benthamiana*, suggesting that PepMoV NIa-Pro functioned as a host TGS suppressor. To our knowledge, this is the first report of plant RNA virus directly suppressing the plant DNA methylation pathway.

The DNA methylation in plants is not a static pathway and is dynamically modulated with three steps of establishing DNA methylation, maintenance methylation, and demethylation. Three types of cytosine methylation have been extensively characterized in plants: symmetric CG and CHG sites and asymmetric CHH (where H = A, C, or T) sites (Law and Jacobsen, 2010). Plant DNA viruses, such as geminivirus Rep protein, are the main viral proteins that can alter the levels of expression of the plant DNA methyltransferases responsible for the maintenance of symmetric methylation (Rodrıguez-Negrete et al., 2013), and AC5 protein of *mungbean yellow mosaic India virus* (MYMIV) inhibits methylation of cytosine in CHH and CHG to suppress *NbDRM2* expression (Li et al., 2015). These results suggest that plant DNA viruses can modulate the host DNA methylation pathway in different manners. However, evidence of plant RNA viruses interacting with the host DNA methylation pathway was observed in a study where the CMV 1a protein was methylated by a novel protein involving host DNA methylation (Kim et al., 2008); this is not direct evidence of plant RNA viruses with the capacity to modulate host DNA methylation. PepMoV NIa-Pro can reverse established TGS by suppressing *NbAGO4* (Figure 6), which is critical for establishing DNA methylation in the first step of the plant DNA methylation pathway (Law and Jacobsen, 2010). NIa-pro significantly activates the expression of *NbMET1*, *NbDRM2*, and *NbCMT*, which catalyze CG, CHG, and CHH methylation (Law and Jacobsen, 2010), suggesting that NIa-Pro likely activates the second step of plant DNA methylation. Interestingly, NIa-Pro could also activate the expression of *NbROS1* and *NbROS2* (Figure 6), which has been demonstrated to demethylate DNA methylation (Kim et al., 2019). It is likely that PepMoV NIa-Pro also possesses the capacity to inhibit the plant DNA methylation pathway through activating demethylation, which is the last step of plant DNA methylation. Further studies of the molecular mechanisms of PepMoV NIa-Pro modulating the plant DNA methylation pathway would contribute to uncovering novel modulating mechanisms of plant DNA methylation by plant RNA viruses.

Overall, the observations of this study show that PepMoV NIa-Pro is a multifunctional protein, and to the best of our knowledge, this is the first report testifying that a potyvirus NIa-Pro protein can both determine the symptom and induce an HR and modulate plant DNA methylation in multiple manners.

## Conclusion

In conclusion, the results of this study suggest that PepMoV NIa-Pro is a pathogenicity determinant and a potent suppressor of host TGS and suggest that NIa-Pro may employ novel mechanisms to suppress host antiviral defenses.

## Data Availability Statement

The raw data supporting the conclusions of this article will be made available by the authors, without undue reservation, to any qualified researcher.

## Author Contributions

S-BZ, X-WL, and YL designed the research and prepared the draft of the manuscript. Y-NG, R-QT, YZ, JP, and OX performed the experiments. Z-HZ, D-YZ, and HL collected the data. All authors reviewed and approved the final version of the manuscript.

## Conflict of Interest

HL is employed by National Agro-Tech Extension and Service Center. The remaining authors declare that the research was conducted in the absence of any commercial or financial relationships that could be construed as a potential conflict of interest.
