# Supplementary material for: The NIa-Protease Protein Encoded by the Pepper Mottle Virus Is a Pathogenicity Determinant and Releases DNA Methylation of Nicotiana benthamiana
Source: Front Microbiol. 2020 Feb 21;11:102. doi: 10.3389/fmicb.2020.00102 (PMC7047827; doi:10.3389/fmicb.2020.00102)
Supplement: Supplementary file 1 [file Data_Sheet_1.PDF]

Supporting Materials

Table S1

Table S1 Primers and Probe used in this study

Primer/Probe	Sequence*	Purpose
PepMoV/NIa-Pro/ClaI/F	5'-CCATCGATATGGCGAAAACTTTGATGAGG-3'	pPVX-NIa-Pro, for over-expression of NIa-Pro via pGR106 (PVX) vector
PepMoV/NIa-Pro/SalI/R	5'-ACGCGTCGACTTGTTCCCTCACACTTTCACTC-3'	pPVX-NIa-Pro, for over-expression of NIa-Pro via pGR106 (PVX) vector
GAPDH/RT-qPCR/F	5'-GCAGTGAACGACCCATTTATCTC-3'	Relative RT-qPCR analysis of NbGADPH
GAPDH/RT-qPCR/R	5'-AACCTTCTTGGCACCACCCT-3'	Relative RT-qPCR analysis of NbGADPH
NbMET1/RT-qPCR/F	5'-TGAATAGTTTGCCACTGCCAGGAC-3'	Relative RT-qPCR analysis of NbMET1
NbMET1/RT-qPCR/R	5'-AGCGAAATGTTTGTTTTGGTTGA-3'	Relative RT-qPCR analysis of NbMET1
NbDRM2/RT-qPCR/F	5'-AAGGCAAAGGCGATTTTTGAGGAAG-3'	Relative RT-qPCR analysis of NbDRM2
NbDRM2/RT-qPCR/R	5'-CTTTGTCCGTGGATCCCAAGATG-3'	Relative RT-qPCR analysis of NbDRM2
NbCMT3/RT-qPCR/F	5'-CTTACGGTCTTCCACAGTTTCG-3'	Relative RT-qPCR analysis of NbCMT3

NbCMT3/RT-qPCR/R	5'-CCATTTCATCTCTTTGCTCATT-3'	Relative RT-qPCR analysis of NbCMT3
NbROS1/RT-qPCR/F	5'-GAAGGAACTGAATCAAGCAACT-3'	Relative RT-qPCR analysis of NbROS1
NbROS1/RT-qPCR/R	5'-GGCATCAGACATAAGTCCAAAT-3'	Relative RT-qPCR analysis of NbROS1
NbROS2/RT-qPCR/F	5'-ATTCAAACGAAGAAAAAACAGC-3'	Relative RT-qPCR analysis of NbROS2
NbROS2/RT-qPCR/R	5'-CAGAACTCGGAGAAGAAGGCAA-3'	Relative RT-qPCR analysis of NbROS2
NbDCL3/RT-qPCR/F	5'-CTACAAGCAGAGAAGGATCATGGAA-3'	Relative RT-qPCR analysis of NbDCL3
NbDCL3/RT-qPCR/R	5'-GAGTCAACAGAGCGTAAATCCAAGT-3'	Relative RT-qPCR analysis of NbDCL3
NbAGO1/RT-qPCR/F	5'-CTGGCGTGGCTTCTATCAAAGTATT-3'	Relative RT-qPCR analysis of NbAGO1
NbAGO1/RT-qPCR/R	5'-CACCTTTACACCTCTCAGTGCCTTC-3'	Relative RT-qPCR analysis of NbAGO1
NbAGO4/RT-qPCR/F	5'-GGAACTATGACTTCTACCTGTGTGCC-3'	Relative RT-qPCR analysis of NbAGO4
NbAGO4/RT-qPCR/R	5'-AACTTCATCCATTGTCCAACTTGTGT-3'	Relative RT-qPCR analysis of NbAGO4
35Spro/F	5'-AAGGYAAGTAATAGAGATTGGATG-3'	Relative qPCR analysis of 35S promoter

35Spro/R	5'-CACCTTCCTTTTCCACTATCTTCACA-3'	Relative qPCR analysis of 35S promoter
GFP/F	5'-CATGAGTAAAGGAGAAGAACTTTTC-3'	Relative qPCR analysis of GFP
GFP/R	5'-TTCATATGATCTGGGTATCTTG-3'	Relative qPCR analysis of GFP
NbActin/F	5'-CACCACAACAGCAGAGCGGGA-3'	Relative qPCR analysis of NbActin
NbActin/R	5'-TCCCACAAACGAGGGCTGGA-3'	Relative qPCR analysis of NbActin

*Restriction sites are underlined.
